# Student Burnout: A Review on Factors Contributing to Burnout Across Different Student Populations

**DOI:** 10.3390/bs15020170

**Published:** 2025-02-05

**Authors:** Liang Zhe Chong, Lee Kien Foo, Sook-Ling Chua

**Affiliations:** Faculty of Computing and Informatics, Multimedia University, Persiaran Multimedia, Cyberjaya 63100, Malaysiaslchua@mmu.edu.my (S.-L.C.)

**Keywords:** burnout, Maslach burnout inventory, student, survey

## Abstract

Burnout refers to a process of chronic response to stress in life. For students, burnout can be caused by the stress related to their study. There are many factors that contribute to burnout in different student populations. The objective of this paper is to review the studies that investigated the factors contributing to burnout in various student populations. Following the Preferred Reporting Items for Systematic Reviews and Meta-Analyses guidelines, we selected 38 recent studies, published between 2019 and 2024. The findings of this review outline the study design, burnout questionnaire, factors investigated, and analysis methods employed in the literature. We further discuss the main factors contributing to student burnout and propose ways to mitigate this issue.

## 1. Introduction

Over the last decade, students across the world have been experiencing burnout. The effects of burnout can have a significant impact on their mental health and impede their academic performance. Burnout refers to a process of chronic response to occupational stress (Maslach & Jackson, 1981). The definition of burnout is further expanded to three key dimensions: exhaustion, cynicism, and inefficacy (Leiter & Schaufeli, 1996). Students experiencing burnout typically feel exhausted, cynical, and lack efficacy due to the compulsion to study, pessimism toward homework assignments or examinations, and low personal accomplishment (Rahmatpour et al., 2019).

The COVID-19 pandemic has further disrupted and exacerbated the educational routines of many students, resulting in an increased incidence of burnout and school avoidance (Gao, 2023). According to Gao (2023), a survey conducted by the China Care for the Next Generation Working Committee in 2020 revealed that over 70% of adolescents experienced academic burnout. Findings from previous studies have shown that academic burnout has a negative impact on students’ academic performance and affects their mental health, including inducing feelings of stress, anxiety, frustration, and fear (Galbraith & Merrill, 2014; Liu et al., 2024; Pham Thi & Duong, 2024; Sun et al., 2024). These may result in additional financial burden on students and delayed graduation from having to retake their failed courses.

Motivated by these, our aim in this paper is to identify key factors contributing to burnout across different student populations including adolescent students, medical students, and those in tertiary education. This paper examines studies published from 2019 to 2024 within the following rational aspects of burnout:

- What factors have been investigated by the authors of previous studies?
- What study design was employed?
- Which burnout questionnaire was administered?
- What are the methods employed for analysis?

The remainder of this paper is structured as follows: Section 2 discusses the methodology in conducting the literature search; Section 3 presents the findings; Section 4 presents the discussions; and Section 5 summarizes the work conducted in this study.

## 2. Methodology

This section describes the methodology employed in carrying out the literature search.

### 2.1. Search Strategy

A literature review was conducted on studies published between 2019 and 2024, following the Preferred Reporting Items for Systematic Reviews and Meta-Analyses (PRISMA) guidelines (Page et al., 2021). The papers were searched from Google Scholar, Semantic Scholar and Inciteful.xyz. We used the following search terms in various combinations: “students”, “burnout”, “engagement”, “youth”, “young people”, “adolescent”, “university” and “mental health”. Snowball strategy was employed to review references within identified articles, allowing for the inclusion of relevant additional articles. Articles irrespective of the country of origin or study design were considered for inclusion.

### 2.2. Eligibility Criteria

The inclusion criteria for papers in this review are as follows: (1) papers published between the years 2019 and 2024, (2) works involving students as participants, and (3) studies investigating burnout. The literature type was limited to journal articles that are not review papers.

### 2.3. Selection Process

**Figure 1:**
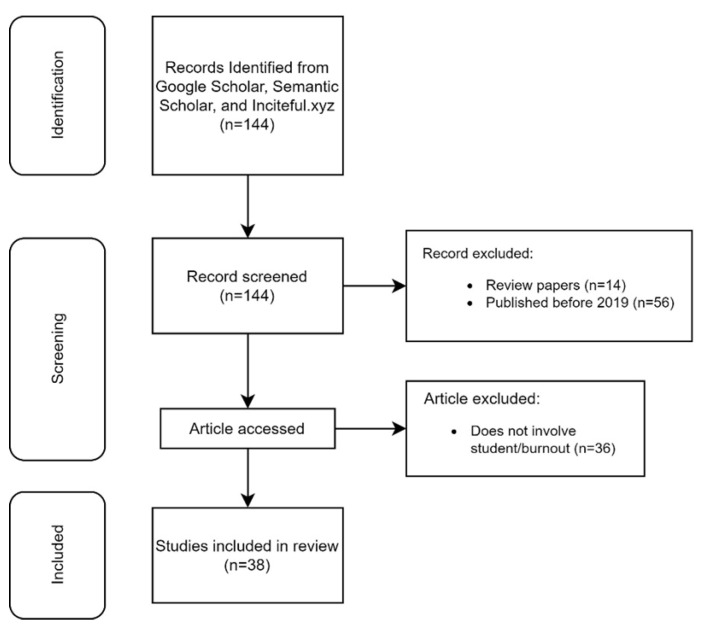
Study selection flow chart.

From the initial search, 144 studies were identified. Upon reviewing the titles and abstracts, 14 papers were excluded because they were review papers, while 56 papers were eliminated for being published before 2019. Another 36 papers were excluded as these papers either focused on non-student populations or did not involve burnout. As a result, 38 studies met the criteria for inclusion and were included in the final review. Figure 1 outlines the study selection process.

## 3. Findings from Reviewed Studies

We have stratified the 38 studies according to education level and country, as summarised in Table 1. We have also included the sample size for each study.

The findings from the literature were reviewed based on the following areas: (1) factors affecting burnout, (2) study design, (3) instruments used to measure burnout, and (4) methods for data analysis.

### 3.1. Factors Affecting Burnout

Various factors were found to be related to student burnout, ranging from environmental influences and psychological traits to personal characteristics. These factors are discussed in the following subsections.

#### 3.1.1. Environmental Factors

**School Environment** School environment was found to be an important determinant of student burnout level. A survey conducted on 364 secondary school students in Turkey showed a negative relationship between quality of school life (QSL) and burnout, with higher QSL related to lower level of burnouts (Gündogan & Özgen, 2020). The QSL in the study covered five aspects of students’ experiences in school: (1) students’ perceptions of their teachers; (2) relationships among students; (3) students’ attitudes toward their school, such as sense of belonging and satisfaction with the school experience; (4) students’ perception of the school management; and (5) students’ feelings of self-worth and the importance accorded to them by others within the school. Similarly, in a cross-sectional study of 550 secondary school students in Turkey, Yildiz and Kiliç (2020) investigated the relationship between school burnout and school attachment. School attachment in this study refers to the positive attitudes of students such as attendance, participation, effort, and psychological connections with the school environment. The study found that lower feelings of success and higher emotional exhaustion resulted in lower students’ attachment to school. Collectively, the findings from these studies indicate that a healthy school environment could reduce burnout among students.
**Impact of Digital Technologies** Excessive use of the internet in young people is recognised as a risk factor to burnout. In a cross-sectional study conducted among 230 high school students in Poland, Tomaszek and Muchacka-Cymerman (2020) found that higher levels of school burnout were significantly associated with higher levels of internet usage. Specifically, burnout from studying, loss of interest in school, and burnout due to parental pressure were key predictors of internet addiction. Similarly, a longitudinal study involving 115 university students found that there is a significant increase in cynicism with Facebook addiction (Tomaszek & Muchacka-Cymerman, 2021).
  The COVID-19 pandemic has further exacerbated the situation by accelerating the shift toward digital learning. While digital learning allows for flexibility compared to traditional classroom learning, it has undoubtedly contributed to feelings of isolation and increased burnout. A longitudinal study was conducted during the COVID-19 pandemic on 680 university students in the Netherlands. The study examined how emergency remote teaching impacted burnout levels (Vollmann et al., 2022). The findings from the study showed that students’ burnout level significantly increased during periods of full-campus closure compared to partial reopening. Similarly, a cross-sectional study of 206 Chinese university students found that inappropriate and excessive use of digital teaching technologies was positively correlated with burnout among students (Song et al., 2022). The over-reliance on smartphones for both academic and non-academic purposes during class was linked to reduced learning motivation. Collectively, the above findings indicate the double-edged sword nature of digital/internet technologies, which, when used inappropriately, can negatively impact student well-being and lead to burnout.
**Social and Family Support** The presence of strong social and family support can help reduce the risk of students experiencing burnout. A study involving 866 high school students from various schools in Turkey found that increased family involvement is associated with reduced levels of school burnout in students (Özhan & Yüksel, 2021). The findings from a cross-sectional study of 1,073 Spanish medical students found that family support was associated with lower burnout levels across all three dimensions of the Maslach Burnout Inventory (Gil-Calderón et al., 2021).
  In a cross-sectional study involving 684 Chinese medical students, the relationship between social support and burnout was evaluated (Zhang et al., 2021). The study reported that high burnout levels were associated with lower perceived social support, particularly subjective support (e.g., feelings of being respected and understood) and the utilization of support (e.g., seeking help from others). Similarly, in a cross-sectional study involving 342 medical students from different academic years in Belgium, perceived social support was found to be a consistent protective factor across all domains of burnout (Kilic et al., 2021).
  In a longitudinal study of 116 online college students, Huang et al. (2023) examined how social support and cognitive engagement influence burnout. The study found that while receiving social support did not directly reduce learning burnout, it positively impacted cognitive engagement, which in turn helped reduce burnout. The evidence from these studies suggests that fostering social and family support are key strategies to mitigate burnout among students. Educators should focus on ensuring that students feel supported to help students manage the demands of education effectively.

#### 3.1.2. Psychosocial Factors

**Psychosocial Competencies** The lack of psychosocial competencies increases the risk of students developing burnout symptoms. In a cross-sectional study conducted among 569 primary school students in Spain, positive relationship was found between expressive suppression (an emotional regulation strategy) and burnout dimensions (cynicism and exhaustion) in students who received weekly tutoring that was designed to help them in emotional regulation (Chacón-Cuberos et al., 2019). These relationships were not significant in students who did not receive tutoring.
  In a cross-sectional study of 1038 high school students in Finland, students who reported higher curiosity, grit, academic buoyancy, social engagement, and sense of belonging were more likely associated with the engaged group than the stressed or burned-out groups (Salmela-Aro & Upadyaya, 2020). This finding is consistent with the study of (Farina et al., 2020), which examined whether empathy has an impact on student burnout. The cross-sectional study was conducted on 998 high school students in Italy. The authors found that empathy is associated with students’ satisfaction in school relations (relations with peers and teachers) and school burnout. The students’ satisfaction in school relations reduced the risk of burnout. The affective component in empathy, on the other hand, has a positive effect on the level of burnout. Similarly, a survey of 1287 high school students in Spain found that components of emotional intelligence, in particular, stress management and mood, fully mediated the negative relationship between academic performance with burnout, suggesting that enhancing emotional intelligence could help prevent burnout (del Jurado et al., 2021).
  Jumat et al. (2020) reported that grit is associated with the absence of burnout. A one-unit increase in grit score was linked to a 19% average increase in the odds of not experiencing burnout. Furthermore, Burr and Beck Dallaghan (2019) found that positive emotional traits such as hope and pride, along with professional efficacy, can help to improve academic performance and mitigate burnout in medical students. Similarly, a longitudinal study involving 135 undergraduate psychology students from a Romanian university found that higher self-efficacy was associated with lower burnout and higher engagement (Maricuțoiu & Sulea, 2019).
  Higher scores on the Strength of Motivation for Medical School (SMMS) questionnaire were also associated with lower burnout, suggesting that positive and optimistic attitudes can help to combat burnout among students (Obregon et al., 2020). Likewise, in a cross-sectional study of 2882 students from a university in Germany, students who actively enhance their resources and seek challenging demands (e.g., volunteering for complex projects, participating in research, taking on leadership roles in student organizations) experience lower levels of exhaustion (Mülder et al., 2022). On the contrary, individuals with high neuroticism are more likely to feel anxious, depressed and emotionally unstable (Kendler et al., 2004; Widiger & Oltmanns, 2017). They often perceive ordinary situations as threatening and minor frustrations as hopelessly difficult. In a cross-sectional study involving 378 public university students in Latin America, students who reported high levels of neuroticism experienced high levels of academic burnout (Ramirez-Asis et al., 2023). Collectively, these studies highlight the importance of psychosocial competencies to combat against school burnout and foster school engagement among students.
**Coping Strategies** Employing appropriate coping strategies can effectively reduce the burnout experienced by students. In a cross-sectional study involving 532 Spanish undergraduate students, Vizoso et al. (2019) found that academic burnout was positively associated with maladaptive coping strategies (e.g., problem avoidance, self-criticism, wishful thinking, and social withdrawal) and negatively associated with adaptive coping strategies (e.g., problem-solving, cognitive restructuring, social support, and expressing emotions). Students who relied on maladaptive coping strategies were more likely to experience higher levels of emotional exhaustion and cynicism, and lower levels of academic efficacy. On the other hand, students who employed adaptive coping strategies reported lower levels of emotional exhaustion and cynicism, and higher levels of academic efficacy.
  A cross-sectional study conducted on four medical schools in China revealed that students who are better at coping with stress tend to have lower burnout levels (Xie et al., 2019). Findings from (Wang et al., 2020) highlighted that active coping, characterised by proactive and problem-focused strategies, was found to mitigate the negative effects of poor sleep quality on burnout. Conversely, passive coping, such as avoidance and disengagement, did not significantly mediate the relationship between sleep quality and burnout, indicating that passive coping strategies are less effective in preventing burnout. Vinter (2021) conducted a longitudinal study on 148 Estonian middle school students. The study used latent profile analysis to identify burnout profiles, i.e., “above-average burnout” and “below-average burnout”. Students in the “above-average burnout” profile used maladaptive coping strategies (self-blame, rumination, catastrophising) more frequently than those in the “below-average burnout” profile. In contrast, adaptive coping strategies such as positive reappraisal were used less frequently by the “above-average burnout” group. These studies suggest that applying the correct coping strategies can help to reduce the burnout level among students.

#### 3.1.3. Other Factors

**Academic Performance** Mental burden is known to negatively impact academic performance. The Programme for International Student Assessment (PISA) conducted by the Organization for Economic Co-operation and Development (OECD) revealed that anxiety about schoolwork, homework, and tests is negatively related to performance (OECD, 2017). Findings from (Vizoso et al., 2019) showed that cynicism and reduced academic efficacy were linked to lower academic performance. This finding is consistent with another cross-sectional study conducted on 2652 secondary school students in Spain, which reported that academic efficacy are significant predictors of academic performance (Usán Supervía & Salavera Bordás, 2020). A study involving 866 high school students from various schools in Turkey found that school burnout significantly resulted in lower academic achievement (Özhan & Yüksel, 2021).
  Another study conducted on 519 first-year life science students from a Finnish university found that students in the “exhausted and inefficacious” group reported lower GPA and study credits compared to students in the “interested not burned-out” group (Asikainen et al., 2022). In a two-wave study involving 142 psychology undergraduate students in Romania, Paloș et al. (2019) reported that academic grades could be considered antecedents to both student engagement and burnout. Students with high academic grades has a higher level of engagement and lower level of burnout. The results from this study are consistent with the findings in (Njim et al., 2019), where a lower cumulative GPA was associated with a higher burnout among students. In a cross-sectional study of 651 medical students and residents at a medical school in Nepal, Pokhrel et al. (2020) found that students’ perceptions of their own academic performance were significantly related to burnout. Dissatisfaction with academic performance was associated with higher levels of burnout, depression, and anxiety. Findings from these studies clearly indicate a connection between student burnout and academic outcomes. It is therefore important for educators to understand the factors that contribute to student burnout and help to create a supportive learning environment.
**Demographics**  Herrmann et al. (2019) uncovered significant differences between gender in burnout experiences in a study involving 649 ninth-grade students (59% female) from six academic track schools in Germany. They found that girls reported higher levels of exhaustion compared to boys regardless of academic achievement. However, there is no significant gender differences for the cynicism component of school burnout. The higher school burnout in girls is attributed to their higher academic contingent self-esteem and a more extrinsic motivational orientation. Similar findings were found in (Gil-Calderón et al., 2021; Obregon et al., 2020), where female students tend to experience a higher rate of burnout than the male students. In a study involving 494 Italian university students, female students showed higher levels of exhaustion, cognitive impairment, and emotional impairment than male students (Fiorilli et al., 2022). Similar findings were found in (Jagodics & Szabó, 2022), where female students scored higher on the emotional exhaustion subscale of the Maslach Burnout Inventory-Student Survey (MBI-SS) compared to male students. Contrary to these studies, Yildiz and Kiliç (2020) found that male students were having a higher level of burnout than the female students among the secondary school students in Turkey. However, findings from the study by (Jumat et al., 2020) showed no correlation between gender and burnout among medical students in Singapore.
  Besides gender differences, Armstrong and Reynolds (2020) reported that black medical students have a higher risk of burnout than other ethnic groups, which may be due to being the first child in their family to attend college, lack of physician family members, or facing financial burdens. There are studies that investigated whether occupational status has a significant influence on student burnout. Fiorilli et al. (2022) found no significant burnout differences between working and non-working students. In the study of (Drăghici & Cazan, 2022), they found that although employed students face the challenges of balancing work and academic responsibilities, their occupational status is not significantly related to academic burnout. Employed students may have developed better time management skills and coping strategies due to the necessity of handling both roles, potentially mitigating the impact of burnout. A cross-sectional study involving 3451 students in Germany also found that students working 10–20 h per week experience less burnout compared to those who do not (Olson et al., 2023). Collectively, these findings suggested that there might be a potential necessity for tailored support programmes for specific demographics among students.
**Lifestyle Choices** Unhealthy lifestyle behaviours are recognised as a significant health risk that can negatively impact students’ overall well-being. In a cross-sectional study of 189 medical students in Cyprus, Nteveros et al. (2020) observed that students experiencing burnout were significantly more likely to report poor sleep quality and suffer from mental health issues. Students who consumed alcohol exhibited higher level of cynicism and lower level of efficacy (Pérez-Fuentes et al., 2021). Additionally, higher engagement levels (the opposite of burnout) were associated with healthier lifestyle choices, such as regular exercise and sufficient sleep, and were negatively related to excessive drug and alcohol use (Agarwal et al., 2020). Similarly, a study involving 228 undergraduate students found that poor sleep quality was positively associated with learning burnout (Wang et al., 2020). These findings suggest that a healthy lifestyle is crucial in combating burnout among students.

### 3.2. Study Design

From the literature (Section 3.1), the majority of the studies employed a cross-sectional study design, while 6 out of the 38 studies employed a longitudinal design (Huang et al., 2023; Jumat et al., 2020; Maricuțoiu & Sulea, 2019; Tomaszek & Muchacka-Cymerman, 2021; Vinter, 2021; Vollmann et al., 2022). This may be because longitudinal studies require significant effort and time than cross-sectional studies.

### 3.3. Measurement Instruments

Most of the studies employed Maslach Burnout Inventory (MBI) to evaluate burnout among students. In total, 18 papers adopted a version of MBI (Burr & Beck Dallaghan, 2019; Gil-Calderón et al., 2021; Jagodics & Szabó, 2022; Jumat et al., 2020; del Jurado et al., 2021; Kilic et al., 2021; Maricuțoiu & Sulea, 2019; Mülder et al., 2022; Nteveros et al., 2020; Obregon et al., 2020; Olson et al., 2023; Özhan & Yüksel, 2021; Paloș et al., 2019; Ramirez-Asis et al., 2023; Tomaszek & Muchacka-Cymerman, 2021; Usán Supervía & Salavera Bordás, 2020; Vizoso et al., 2019; Xie et al., 2019). The School Burnout Inventory (SBI) was the second most commonly applied instrument among the reviewed papers, with six papers employing SBI in their studies (Chacón-Cuberos et al., 2019; Farina et al., 2020; Gündogan & Özgen, 2020; Herrmann et al., 2019; Salmela-Aro & Upadyaya, 2020; Vinter, 2021).

Other instruments include the Copenhagen Burnout Inventory (Armstrong & Reynolds, 2020; Pokhrel et al., 2020) and the Burnout Assessment Tool (Drăghici & Cazan, 2022; Fiorilli et al., 2022). Besides MBI and SBI, there are studies that applied alternative instruments such as the Utrecht Burnout Scale for Students (Vollmann et al., 2022), Oldenburg Burnout Inventory (Njim et al., 2019), Learning Burnout Scale (Zhang et al., 2021), Burnout Measure Short Version (Agarwal et al., 2020), School Burnout Scale (Yildiz & Kiliç, 2020), Student School Burnout Scale (Tomaszek & Muchacka-Cymerman, 2021), Study Burnout Inventory (Asikainen et al., 2022), Classroom Burnout Inventory (Song et al., 2022), Learning Burnout Questionnaire (Wang et al., 2020), and the Academic Burnout Scale (Huang et al., 2023).

### 3.4. Data Analysis Method

Structural equation modelling (SEM) was found to be popular among the reviewed papers. Thirteen papers employed SEM to analyse relationships between variables (Chacón-Cuberos et al., 2019; Farina et al., 2020; Gündogan & Özgen, 2020; Herrmann et al., 2019; Huang et al., 2023; Jagodics & Szabó, 2022; Maricuțoiu & Sulea, 2019; Özhan & Yüksel, 2021; Paloș et al., 2019; Ramirez-Asis et al., 2023; Usán Supervía & Salavera Bordás, 2020; Vizoso et al., 2019; Wang et al., 2020). Linear regression analysis is also a popular method, with 10 studies utilising linear regression analysis (Burr & Beck Dallaghan, 2019; Gil-Calderón et al., 2021; Kilic et al., 2021; Njim et al., 2019; Obregon et al., 2020; Song et al., 2022; Tomaszek & Muchacka-Cymerman, 2020, 2021; Xie et al., 2019; Yildiz & Kiliç, 2020).

Logistic regressions were employed in five studies (Armstrong & Reynolds, 2020; Jumat et al., 2020; Olson et al., 2023; Pokhrel et al., 2020; Zhang et al., 2021). Latent profile analysis was used in four studies (Asikainen et al., 2022; Mülder et al., 2022; Salmela-Aro & Upadyaya, 2020; Vinter, 2021). Analysis of variance was employed in three studies (Drăghici & Cazan, 2022; Fiorilli et al., 2022; Vollmann et al., 2022), while correlation analysis was carried out in two studies (Agarwal et al., 2020; del Jurado et al., 2021). Mann–Whitney U test was employed in one study (Nteveros et al., 2020).

## 4. Discussion

This paper reviewed studies from different nations involving students across diverse educational backgrounds. The findings from our review indicate the following:

1. A supportive school environment is crucial in mitigating student burnout. Poor school attachment is positively associated to higher burnout among students, suggesting the need for educational institutions to foster an environment that supports students’ psychological and emotional needs.
2. Psychosocial competencies play a vital role in preventing burnout. Schools should focus not only on the academic aspect but also psychosocial aspects of students. Appropriate emotional regulation strategies and positive traits such as curiosity, grit, and social engagement can help students remain engaged and less prone to burnout.
3. Problematic internet use is identified as a significant risk factor for student burnout. Increased digital engagement has amplified feelings of isolation. As digital environments become increasingly prevalent, there is a pressing need to develop strategies in helping students manage their online activities such as teaching students about healthy internet habits and the risks of excessive use.
4. Strong social and family support creates a protective barrier against burnout. Studies indicate that perceived social support, such as family involvement and feelings of being respected reduces burnout levels. Institutions and parents should prioritize creating support networks to help students cope with academic and emotional challenges.
5. Effective coping strategies are crucial in mitigating burnout. Adaptive strategies such as problem-solving and social support are associated with lower burnout levels, while maladaptive strategies such as avoidance and self-criticism increase burnout. The findings from the literature suggest that there is a need for guiding students with proper coping mechanisms to handle burnout.
6. There is an association between burnout and academic performance. High levels of cynicism and reduced academic efficacy are related to lower academic achievement. Addressing student burnout may be a more effective approach to improving academic performance, instead of focusing solely on students’ academic work without addressing the underlying root causes.
7. Some demographic groups are prone to burnout. For instance, female students often have higher exhaustion levels than male students. Ethnic background also influences burnout risk, with black medical students facing higher burnout due to additional stressors such as being first-generation college students. Tailored support programs for specific demographic needs might be beneficial in addressing burnout for different demographic groups.
8. Unhealthy lifestyle choices are linked to higher levels of stress and burnout. Poor sleep quality and excessive use of drug and alcohol worsen burnout. Healthy lifestyle choices with regular exercise and adequate sleep can help in preventing burnout among students.

## 5. Conclusions

This paper reviewed the current state of research on student burnout. We started by introducing the prevalence of burnout, describing our scope and outlining the research questions. Next, we discussed our methods in the literature search. Then, we presented the findings, revealing the factors contributing to student burnout. We also summarised the study designs, the measurement instruments used, and the analysis employed in current studies. Our findings revealed that student burnout is a complex issue, influenced by a variety of factors. A supportive school environment that encourages the development of psychosocial needs, positive social and familial support, the adoption of adaptive coping strategies, and the maintenance of a healthy lifestyle can help in lowering the risk of burnout. All these require collaborative effort from educational institutions, families, and policy makers for an effective mitigation strategy.

## Figures and Tables

**Table 1:** Grouping of studies based on education level and country.

Education Level	Country	Studies	Sample Size
Primary School	Spain	Chacón-Cuberos et al. (2019)	569
Secondary/High school	Germany	Herrmann et al. (2019)	649
	Estonia	Vinter (2021)	148
	Finland	Salmela-Aro and Upadyaya (2020)	1038
	Italy	Farina et al. (2020)	998
	Poland	Tomaszek and Muchacka-Cymerman (2020)	230
	Spain	del Jurado et al. (2021)	1287
		Usán Supervía and Salavera Bordás (2020)	2652
	Turkey	Gündogan and Özgen (2020)	364
		Yildiz and Kiliç (2020)	550
		Özhan and Yüksel (2021)	866
University/College	Belgium	Kilic et al. (2021)	342
	Cameroon	Njim et al. (2019)	413
	China	Xie et al. (2019)	1977
		Wang et al. (2020)	228
		Zhang et al. (2021)	684
		Song et al. (2022)	206
		Huang et al. (2023)	116
	Cyprus	Nteveros et al. (2020)	189
	Finland	Asikainen et al. (2022)	519
	Germany	Mülder et al. (2022)	2882
		Olson et al. (2023)	3451
	Hungary	Jagodics and Szabó (2022)	743
	Italy	Fiorilli et al. (2022)	494
	Latin America	Ramirez-Asis et al. (2023)	378
	Nepal	Pokhrel et al. (2020)	651
	The Netherlands	Vollmann et al. (2022)	680
	Poland	Tomaszek and Muchacka-Cymerman (2021)	115
	Romania	Maricuțoiu and Sulea (2019)	135
		Paloș et al. (2019)	142
		Drăghici and Cazan (2022)	151
	Singapore	Jumat et al. (2020)	59
	Spain	Vizoso et al. (2019)	532
		Gil-Calderón et al. (2021)	1073
	United States	Burr and Beck Dallaghan (2019)	264
		Agarwal et al. (2020)	287
		Armstrong and Reynolds (2020)	162
		Obregon et al. (2020)	273

## Data Availability

No new data were created or analysed in this study. Data sharing is not applicable to this article.
